# Neuron-Specific Enolase (NSE) Predicts Long-Term Mortality in Adult Patients after Cardiac Arrest: Results from a Prospective Trial

**DOI:** 10.3390/medicines8110072

**Published:** 2021-11-17

**Authors:** Jonas Müller, Benjamin Bissmann, Christoph Becker, Katharina Beck, Nina Loretz, Sebastian Gross, Simon A. Amacher, Chantal Bohren, Hans Pargger, Kai Tisljar, Raoul Sutter, Stephan Marsch, Sabina Hunziker

**Affiliations:** 1 Department of Medical Communication and Psychosomatic Medicine, University Hospital Basel, 4031 Basel, Switzerland; jonasfabian.mueller@usb.ch (J.M.); benjamin.bissmann@usb.ch (B.B.); christoph.becker@usb.ch (C.B.); juliakatharina.beck@usb.ch (K.B.); ninachiara.loretz@usb.ch (N.L.); sebastianseverin.gross@usb.ch (S.G.); simonadrian.amacher@usb.ch (S.A.A.); chantalbeatrice.bohren@usb.ch (C.B.); 2 Emergency Department, University Hospital Basel, 4031 Basel, Switzerland; 3 Medical Faculty, University of Basel, 4031 Basel, Switzerland; hans.pargger@usb.ch (H.P.); kai.tisljar@usb.ch (K.T.); raoul.sutter@usb.ch (R.S.); stephan.marsch@usb.ch (S.M.); 4 Intensive Care Unit, University Hospital Basel, 4031 Basel, Switzerland

**Keywords:** cardiac arrest, cardiopulmonary resuscitation, neuron-specific enolase, prognostication, long-term mortality, neurological outcome

## Abstract

Background: We investigated whether Neuron-specific enolase (NSE) serum concentration predicts long-term mortality and poor neurological outcome in adult cardiac arrest patients. Methods: Within this prospective observational study, we included consecutive adult patients admitted to the intensive care unit (ICU) after cardiac arrest. NSE was measured upon ICU admission and on days 1, 2, 3, 5 and 7. Results: Of 403 patients, 176 (43.7%) survived. Median follow-up duration was 43.7 months (IQR 14.3 to 63.0 months). NSE levels on day 3 were increased more than threefold in non-survivors compared to survivors (median NSE (ng/mL) 19.8 (IQR 15.7 to 27.8) vs. 72.6 (IQR 26 to 194)) and showed the highest prognostic performance for mortality compared to other days of measurement, with an AUC of 0.81 and an adjusted hazard ratio of 1.55 (95% CI 1.41 to 1.71, *p* < 0.001). Subgroup analysis showed an excellent sensitivity and negative predictive value of 100% of NSE in patients <54 years of age. Conclusion: NSE measured three days after cardiac arrest is associated with long-term mortality and neurological outcome and may provide prognostic information that improves clinical decision making. Particularly in the subgroup of younger patients (<54 years), NSE showed excellent negative predictive value.

## 1. Introduction

Mortality and risk for poor neurological outcome in adult patients after cardiac arrest has improved but remains high, despite advances in prehospital, emergency and intensive care medicine [1,2]. In these patients, early risk stratification tools might help clinicians and relatives as decision makers to choose further therapeutic approaches and to decide whether to escalate or withdraw from therapy, in alignment with the patients presumed will. However, the prediction of outcome after cardiac arrest is challenging, especially in patients undergoing a targeted temperature management, which often requires sedation and relaxation [3]. Several clinical studies have focused on predicting short-term mortality following cardiac arrest, intending to improve prognostication and potentially influencing decision making regarding therapeutic options [4]. Recently, a study demonstrated that different routine blood markers were beneficial in prognostication of outcome of cardiac arrest patients [5]; in particular, levels of inflammation and shock correlated with poor outcome. Yet, there has been little research looking at long-term outcomes in patients surviving after cardiac arrest. Understanding risk factors for long-term prognosis may also guide caregivers’ and families’ decision making in the acute phase.

Research so far has indicated that cardiac arrest survivors face higher overall mortality and morbidity than the average population [6], especially younger patients [2]. Cardiac arrests predominantly occur in people with significant comorbidities [7]. However, cardiac arrests can have severe neurological and psychological sequelae that impair quality of daily living and predispose individuals to functional impairment, loss of independence, clinical deterioration and death [2,6,8,9].

To improve short-term prognostication, international guidelines recommend the measurement of neuron-specific enolase (NSE) in addition to neuroimaging and neurophysiologic studies [10,11]. NSE, also known as enolase γ, is a glycolytic enzyme located mainly in neurons and neuroectodermal cells [12]. In healthy individuals, serum levels of NSE remain low. In contrast, upon damage to neuronal tissue, such as from stroke, seizure or anoxic brain injury, NSE serum concentration increases and consequently acts as a biomarker for brain damage [13]. Circulating NSE has a half-life of approximately 30 h [14]. Several systematic reviews conclude that NSE measured between 48–72 h after return of spontaneous circulation (ROSC) provides the most accurate prognostic information regarding mortality and neurological outcome [4,11,15,16]. However, most studies have only looked at short-term outcomes, with studies on longer-term outcomes remaining sparse [17,18,19,20,21,22].

Herein, we aimed to assess whether NSE serum levels would predict long-term outcomes in adult patients after cardiac arrest. We hypothesized that NSE serum concentration measured within the first week after cardiac arrest is associated with long-term mortality as well as poor neurological outcome.

## 2. Material and Methods

### 2.1. Study Setting

In this ongoing prospective observational study [23,24], we included consecutive patients after cardiac arrest from October 2012 to October 2020 at the University Hospital Basel, Switzerland. The local ethics committee (Ethics Committee of Northwest and Central Switzerland, Ethikkomission Nordwest und Zentralschweiz; EKNZ) approved the study. Patients or their relatives provided informed consent for study participation. If no next of kin was readily available, we asked a physician in the treatment team, who was not involved in the study, to confirm the patient’s involvement in the study. In these cases, we asked the patient or next of kin to later confirm the inclusion.

### 2.2. Study Population and Treatment of Patients during the Trial

We enrolled consecutive adult patients (i.e., ≥16 years of age) who experienced a cardiac arrest, achieved return of spontaneous circulation (ROSC) and were admitted to the intensive care unit (ICU) at the University Hospital Basel. We excluded patients with monitored in-hospital cardiac arrest (IHCA) or patients for whom NSE serum levels were not measured as part of the clinical routine.

The treatment of patients regarding cardiac arrest followed the clinical routine in our ICU without interaction with the research team. In 2012, all consecutive patients without complete recovery to premorbid neurofunctional baseline within the first hours following resuscitation were cooled systemically with the thermogard XP temperature management system (ZOLL^®^ Medical Corporation, Chelmsford, MA, USA), as a neuroprotectant measure to a target a core temperature of 34.0 °C (i.e., 93.2 °F) for 24 h, followed by a rewarming phase with a controlled increase of the core temperature (i.e., 0.1 °C or 0.2 °F per hour) to 37.5 °C (i.e., 99.5 °F) [25]. Since 2013, following the TTM-trial [26], all consecutive patients without complete recovery have been cooled to a target core temperature of 36.0 °C (i.e., 96.8 °F) for 28 h, followed by the rewarming phase, using the thermogard XP temperature management system. Patients with core temperatures below the target temperature were rewarmed at a rate of 0.5 °C (i.e., 32.9 °F) per hour to meet the target core temperatures.

### 2.3. Data Collection

The treating ICU nurses drew blood samples for measurement of NSE serum levels at ICU admission (day 0) and on days 1, 2, 3, 5 and 7 using an Electro-Chemi-Luminescent-Immuno-Assay (ECLIA) kit (Roche Diagnostics, Rotkreuz, Switzerland). In addition, we collected other routine blood markers, including arterial lactate levels and arterial pH as a surrogate marker of shock to approximate the severity of hypoxia after cardiac arrest [5] and to compare this aspect to other cardiac arrest cohorts.

Trained research investigators collected resuscitation information (i.e., no-flow time (time from cardiac arrest to start of basic life support (BLS)), low-flow time (time from start of BLS to ROSC), cardiac arrest setting, bystander observing the cardiac arrest, and providing cardiopulmonary resuscitation (CPR) and initial rhythm), cause of arrest, as well as socio-demographic parameters (i.e., age, gender and comorbidities (i.e., coronary artery disease, congestive heart failure, chronic obstructive pulmonary disease (COPD), hypertension, malignant disease, diabetes mellitus, chronic kidney disease, neurological disease)) from the electronic medical records (EMR), using a structured data collection form. We did not collect data regarding neuroimaging, clinical neurological, or neurophysiological exams.

To account for missing data in predictors used in the multivariable analyses, we imputed datasets using multiple imputations by chained equations. We calculated imputations using multiple covariables (i.e., socio-demographics, comorbidities, resuscitation information and vital signs) with the inclusion of main outcomes (death, neurological outcome) in order to reduce bias, as suggested by Sterner et al. [27].

### 2.4. Outcomes

The primary endpoint was long-term overall all-cause mortality over 5 years. The secondary endpoint was neurological outcome at 2 years of follow-up measured by the Cerebral Performance Category (CPC) scale. We assessed CPC prospectively with follow-up phone calls and imputation from public records in the case of death. In accordance with previous studies, we defined no or only minor neurological or psychological deficits (CPC = 1) and moderate disability (CPC = 2) as good neurological outcome and severe disability (CPC = 3), coma or vegetative state (CPC = 4) and death (CPC = 5) as poor neurological outcome [28,29]. Patients admitted after October 2018 were removed from analyses of neurological outcome due to too little follow-up time for secondary outcome assessment.

### 2.5. Statistical Analyses

We characterized the patient cohort with descriptive statistics such as means, medians and inter-quartile ranges (IQRs) for continuous variables and frequencies for binary or categorical variables. For analysis of patient characteristics, we used t-tests for continuous variables and chi-squared tests for binary and categorical variables. We then calculated receiver operating characteristics (ROCs) and corresponding areas under the curve (AUC) to evaluate the discrimination of NSE serum levels.

To assess the association of NSE serum concentrations with outcomes, we conducted univariable and multivariable regression analyses with calculation of hazard ratios (HRs) and their 95% confidence intervals (CIs) for mortality using Cox-regression models for time to event data (i.e., mortality) and logistic regression analyses with odds ratios (ORs) for poor neurological outcome. In two multivariable models, we adjusted for age, gender and comorbidities (i.e., coronary artery disease, COPD, hypertension, diabetes mellitus, chronic kidney disease, malignant disease and neurological disease) as well as age, gender and resuscitation circumstances (i.e., in-hospital, bystander-CPR, time to ROSC, initial rhythm and initial serum lactate levels).

We divided NSE levels into deciles for achieving normal distribution, standardization of HRs and ORs and better comparability. We calculated sensitivity, specificity, positive predictive value (PPV), negative predictive value (NPV), positive and negative likelihood ratios as percentages with 95% CI for each sampling day, as well as maximal NSE serum concentration available; concerning all-cause mortality, calculations were made for different time points (i.e., 6, 12, 18 and 24 months), with NSE measurements at day 3 to understand whether differences in performance exist at different points of follow-up. We estimated optimal cut-off levels of serum NSE at the different time points and for the maximal NSE serum value for our patient cohort according to the Youden index. Finally, to understand whether differences in prognostic performance exist within certain patient groups, we performed predefined subgroup analyses based on gender, age, circumstances of resuscitation (i.e., bystander starting CPR), setting of cardiac arrest (at home, public or IHCA), time until ROSC, initial rhythm, temperature management (hypothermia (TMH) and targeted management (TTM)), comorbidities (coronary artery disease, diabetes mellitus, hypertension) and cause of cardiac arrest.

We considered a *p*-value < 0.05 as statistically significant and used STATA 15.0 (Stata Corporation, College Station, TX, USA) for all statistical analyses.

## 3. Results

### 3.1. Baseline Characteristics

A total of 403 patients were admitted to the ICU of the University Hospital Basel after resuscitation from a cardiac arrest and included in the study. A total of 176 (43.7%) survived, with a median follow-up time of 43.7 months (IQR 14.3 months to 62.9 months). Table 1 shows the baseline characteristics stratified by overall all-cause mortality. The mean age of the cohort was 64.1 years and 70.5% were males. Most patients suffered an out-of-hospital cardiac arrest (OHCA), but in 15.9% (n = 62) the cardiac arrest occurred in hospital (not on a monitor). Non-survivors, compared to survivors, had a higher mean age, were a lower proportion of males and had a higher prevalence of congestive heart failure, COPD, diabetes mellitus, chronic kidney disease, malignant disease and neurologic disease, but a lower prevalence of preexisting coronary artery disease.

### 3.2. Association between NSE Blood Levels and Overall Long-Term Mortality

We compared NSE levels on ICU admission and on days 1, 2, 3, 5 and 7 as well as the maximal NSE value of survivors and non-survivors (Table 2a and Supplementary Table S1). In each case, NSE values were significantly higher in non-survivors. The highest difference was found for day 3, with a median NSE value of 19.8 ng/mL (IQR 15.7 to 27.8) among survivors vs. 72.6 ng/mL (IQR 26 to 194) for non-survivors, resulting in a HR of 1.5 per decile (95% CI 1.38 to 1.64), *p* < 0.001, and high discrimination (ROC AUC 0.81). This was robust in multivariable logistic regression analyses, with an adjusted HR of 1.55 (95% CI 1.41 to 1.71), *p* < 0.001, and 1.51 (95% CI 1.37 to 1.66), *p* < 0.001, for the two models.

**Figure 1:**
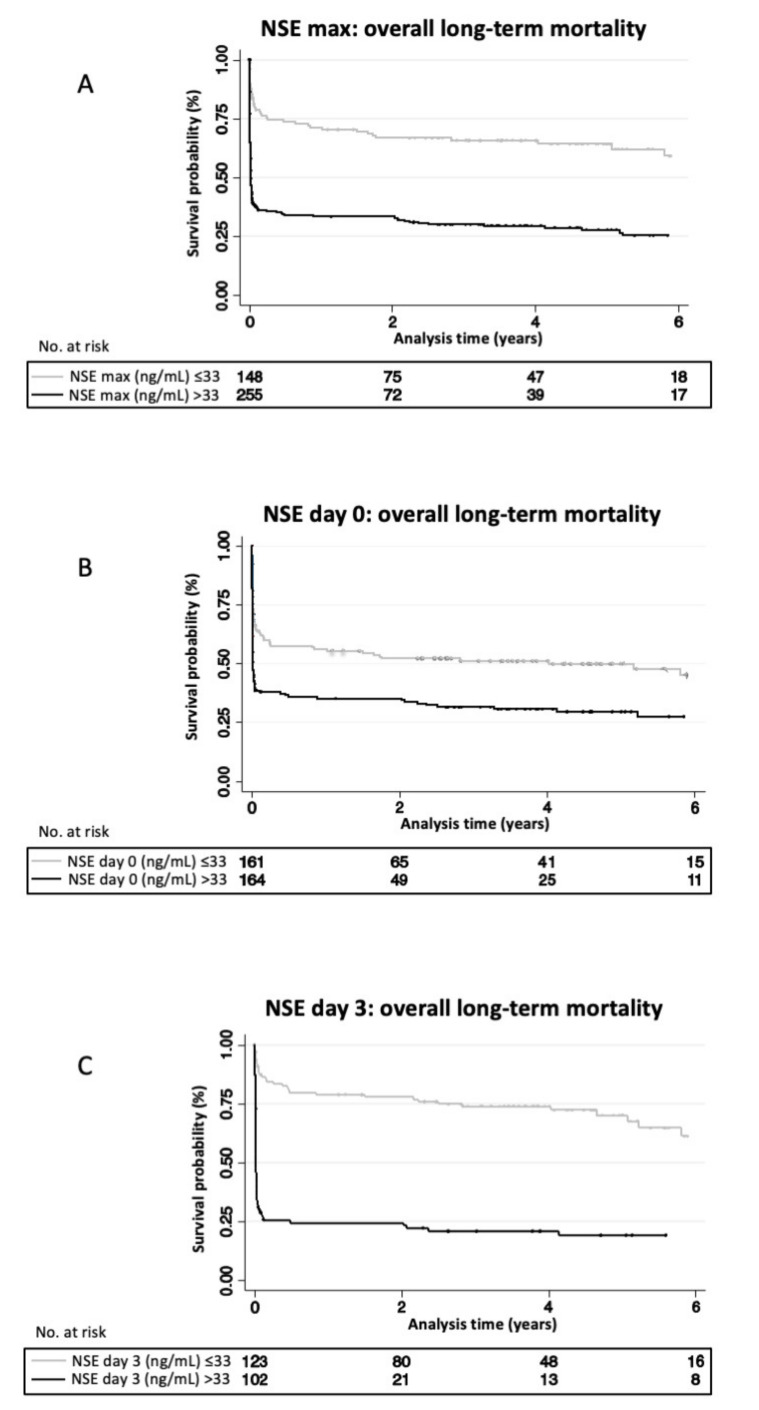
NSE levels associated with overall long-term mortality: Kaplan-Meier curves stratified by serum NSE-concentrations above and below 33ng/mL using maximal NSE measured (**A**), measured on day 0 (**B**) and measured on day 3 (**C**). Log-Rank Test of NSE max *p* < 0.001; Log-Rank Test of NSE day 0 *p* < 0.001; Log-Rank Test of NSE day 3 *p* < 0.001. NSE, neuron specific enolase.

Figure 1 shows Kaplan-Meier curves stratified according to NSE levels on day 0, day 3 and maximal level (cut-off 33 ng/mL). For all NSE levels, survival curves showed a steep decline in survival in the first 2 months and a plateau after 3–6 months. Day 3 NSE values showed the best separation of groups.

Furthermore, we calculated sensitivity and specificity, positive and negative likelihood ratios, and positive and negative predictive values at the cut-off points recommended by the American Academy of Neurology in 2006 (33 ng/mL), as well as for the optimal cut-off based on ROC analysis (Table 3 and Supplementary Table S4) [30]. On day 3, sensitivity and specificity at 33 ng/mL were 70.2% (95% CI 60.9 to 78.4%) and 80.2% (95% CI 71.5 to 87.1%). The calculated optimal cut-off for day 3 was at a threshold of 35.9 ng/mL, with a corresponding sensitivity of 68.4% (95% CI 59.1 to 76.8%) and a specificity of 84.7% (95% CI 76.6 to 90.8%). The performance of NSE values on day 2 at a cut-off of 33 ng/mL was similarly high, with a sensitivity of 68.3% (95% CI 60.0 to 75.7%) and specificity of 81.2% (95% CI 74.0 to 87.1%), and at the calculated optimal cut-off for day 2 (29.5 ng/mL), with a sensitivity of 75.2% (95% CI 67.3 to 82.0%) and a specificity of 73.8% (95% CI 66.0 to 80.7%).

Regarding performance changes with different follow-ups, predictive performance of NSE levels above 33 ng/mL measured at day 3 was similar when observing overall all-cause mortality at 6, 12, 18 and 24 months (Supplementary Table S2).

The performance of NSE regarding overall mortality and neurological outcome was measured on day 3 after cardiac arrest using a threshold of 60 ng/mL, and the specificity was higher (94.6% and 95.2% versus 80.2% and 83.9% for primary and secondary endpoint) at the cost of a lower sensitivity (53.5% and 58.1% at 60 ng/mL versus 70.2% and 75.2% at 33 ng/mL) (Supplementary Table S3).

### 3.3. Association between NSE Blood Levels and Neurological Outcome at 2 Years

At 2 years, 303 patients could be contacted for a follow-up assessment, of which 215 patients (71%) had a poor neurological outcome. Results were similar for neurological outcomes at 2 years, with high NSE levels on day 3 showing the strongest association with poor neurological outcome at 2 year follow-up, with an OR of 1.68 per decile (95% CI 1.43 to 1.96) in univariable logistic regression analysis, and in multivariable logistic regression analyses with an OR of 1.81 per decile (95% CI 1.49 to 2.2) and an OR of 1.67 per decile (95% CI 1.37 to 2.03), respectively (Table 2b and Supplementary Table S1).

Again, NSE levels on day 3 showed the highest performance, with a sensitivity and specificity of 75.2% (95% CI 65.9 to 83.1%) and 83.9% (95% CI 72.3 to 92.0%) at a cut-off of 33 ng/mL and a sensitivity and specificity of 73.3% (95% CI 63.8 to 81.5%) and 87.1% (95% CI 76.1 to 94.3%) at a calculated optimal cut-off (37.1 ng/mL) (Supplementary Table S5).

### 3.4. Subgroup Analyses

**Figure 2:**
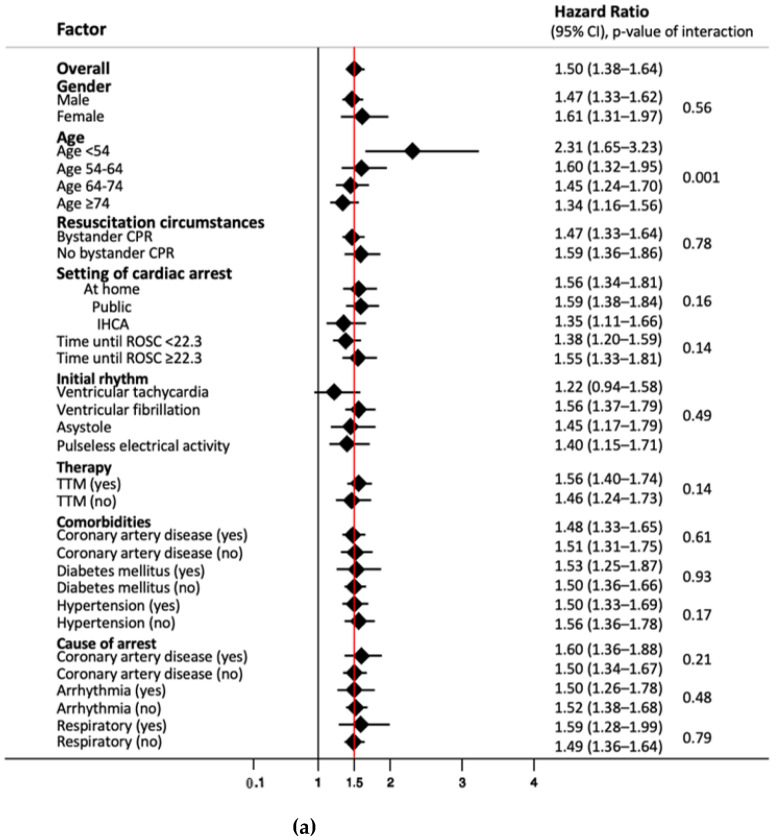

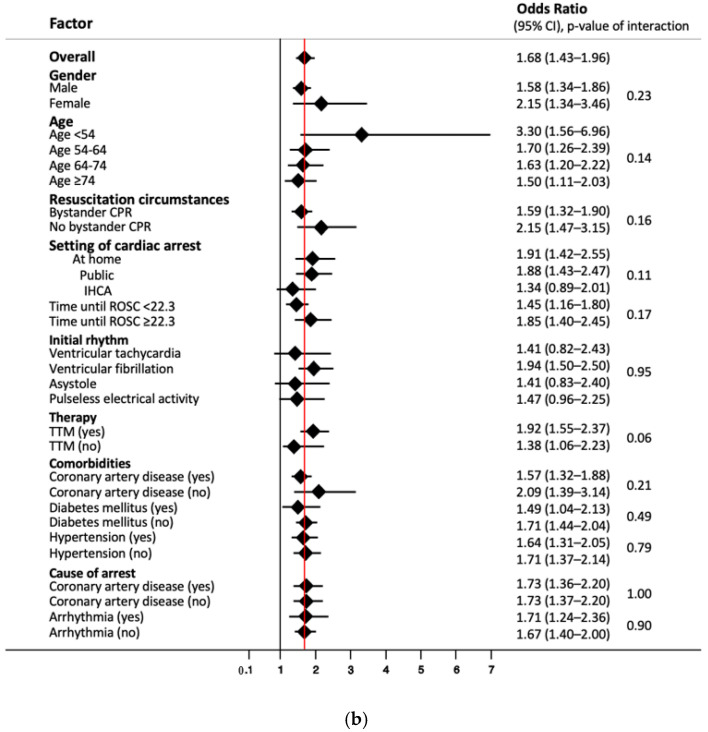
(**a**) Subgroup analysis of NSE levels on day three for endpoint overall long-term mortality. Data presented as hazard ratio (95% confidence interval); CPR, cardiopulmonary resuscitation; IHCA, in-hospital cardiac arrest; ROSC, return of spontaneous circulation; TTM, targeted temperature management. (**b**) Subgroup analysis of NSE levels on day three for endpoint neurological outcome after 2 years. Data presented as odds ratio (95% confidence interval); CPR, cardiopulmonary resuscitation; IHCA, in-hospital cardiac arrest; ROSC, return of spontaneous circulation; TTM, targeted temperature management.

We performed subgroup analyses to look for differences in prognostic value of NSE values on day 3 concerning long-term mortality (Figure 2a) and good/poor neurological outcome after 2 years (Figure 2b). While results were robust in most subgroups, we found that NSE had a significantly better performance in younger patients < 54 years, with a HR of 2.31 compared to the overall HR of 1.5 (*p* of interaction < 0.001). In this subgroup, NSE at a cut-off of 33 ng/mL on day 3 had a sensitivity of 100% (95% CI 83.9 to 100%) and specificity of 80.0% (95% CI 65.4 to 90.4), with corresponding PPV and NPV of 70.0% (95% CI 50.6 to 85.3%) and 100% (95% CI 90.3 to 100%) (Supplementary Table S6). Similarly, NSE showed a better performance for neurological outcome in patients < 54 years without reaching significant interaction analysis results (*p* of interaction 0.14) (Figure 2b).

## 4. Discussion

In this long-term prospective single-center cohort study of adult cardiac arrest patients with a median follow-up of almost 4 years, we found a strong association of NSE serum levels with long-term mortality and neurological outcomes. Similar to known associations with short-term outcomes, day 3 NSE values showed the highest association, with an ROC AUC of 0.81 for mortality and 0.85 for poor neurological outcome. These associations were independent of other prognostic factors in multivariable analyses. While sensitivity and NPV at day 3 were around 70% in the overall population, in younger patients <54 years of age, NSE values showed the best performance, with a sensitivity and NPV of 100% each. Thus, in younger patients, NSE may be beneficial to rule out mortality and adverse neurological outcome and may help to inform initial clinical decision making regarding ruling out of adverse outcomes.

Although long-term outcome studies after cardiac arrest and NSE are sparse, our findings are largely in line with previous studies. Stammet et al. also found associations of NSE serum levels with short-term neurological outcome and mortality in a 3 year follow-up of OHCA patients, which were similar to our long-term results [17]. Another study by Vondrakova et al. [18] including unconscious OHCA patients showed that NSE values on days 3 and 4 and maximal NSE value predicted neurological outcome at 30 days and to a smaller degree mortality at 12 months. Storm et al. found that NSE at 72 h predicted neurological outcome at ICU discharge and to a lesser extent long-term mortality. This was, however, limited to cardiac arrest patients with acute kidney injury [19]. Another study by Wihersaari et al. in OHCA patients found NSE serum values at 48 h predicted long-term neurological outcome at 12 months [20]. In a study with CPR survivors on extracorporeal life support, NSE was correlated with in-hospital mortality and long-term neurological outcome, i.e., CPC after hospital discharge with mean follow-up of 29 +/−24 months [21]. In a study of 89 OHCA patients discharged with unresponsive wakefulness syndrome or coma (CPC 4), where only 5% had an NSE value at 48–72 h below 17 ng/mL, long-term survival was 30% after one year and 11% after 5 years [22]. Our data thus validate these previous studies in an independent prospective cohort of cardiac arrest patients.

The European Resuscitation Council (ERC) highlighted the importance of long-term outcomes in their 2021 guidelines [8]. In addition to the psychosocial impacts, there are long-term health implications after suffering a cardiac arrest [2]. Elevated NSE may act as a surrogate for the severity of hypoxic brain injury [9,13,31,32] and might therefore reflect some of the long-term health consequences. This may be important information for the discussion of prognosis with patients and relatives. Although in this cohort most deaths occurred within three to six months, survival outcomes between patients with higher and lower NSE remained similar over several years. Thus, our findings suggest that the prognostic value of NSE might also be valid for long-term prognosis. However, more research is needed to elucidate the relationship between NSE and long-term outcomes after cardiac arrest.

In our sample, we found the highest prognostic value of NSE serum concentrations when measured on day 3 as compared to days 0, 1, 2, 5, 7 and the maximal value. This is in line with current guidelines recommending NSE measurement between 48 h and 72 h to predict outcome in the short term [4,11,33]. Interestingly, the proposed cut-off of 33 ng/mL again showed fairly good diagnostic measures, although the optimal cut-off according to the Youden index was 35.9 ng/mL in our cohort.

In 2006, the American Academy of Neurology recommended a cut-off of NSE at 33.0 ng/mL [30]. However, this was based on only one study, with NSE samples from 231 cardiac arrest patients [34]. Yet, due to suboptimal performance of NSE and any predictor by itself, the 2021 European Resuscitation Council and European Society of Intensive Care Medicine guidelines advocate for a multimodal approach for neurological prognostication to improve accuracy and reduce the risk for false-negative and false-positive predictions [10]. According to their recommendation, there is minimal risk for false positives when using a higher NSE threshold of >60 ng/mL at 48 h or 72 h in combination with another predictor for poor outcome in comatose patients. However, they also state that thresholds for high serum NSE levels have to be established in collaboration with local laboratories. Another recent systematic review by Wang et al. recommended that institutions should establish institution-specific cut-off thresholds due to the lack of large studies [15]. Our data regarding long-term outcome are in line with these studies, also showing only suboptimal performance of NSE at the 33.0 ng/mL cut-off, with a sensitivity and specificity of around 70–80%, which limits the use of NSE as a single predictor for decision making. However, our data also indicate that in younger patients <54 years of age, serum NSE has an excellent sensitivity and NPV and thus has excellent diagnostic measures to rule out bad outcomes. However, due to the exploratory nature of this subgroup analysis, this finding needs independent external validation in other cohorts.

The better performance of serum NSE concentrations for neurological prognostication in younger cardiac arrest patients has already been described in a previously mentioned Finnish cohort study by Wihersaari et al. that included unconscious OHCA patients and NSE measurements at 48 h [20]. According to their findings, NSE had an overall AUC of 0.72, with an AUC of 0.91 for patients <57 years for poor neurological outcome at 12 months. Similarly, we previously found a trend towards higher prognostic accuracy of NSE for younger patients with respect to short-term neurological outcome at hospital discharge and in-hospital mortality [23]. Yet, another large retrospective multicenter study did not find a better performance of NSE in younger individuals [31]. This study, however, only looked at short-term neurological outcome, i.e., CPC at ICU discharge.

Clearly, there is need for more research looking at the influence of age on the prognostic value of NSE for short- and long-term outcomes in order to understand how to most efficiently use this marker in clinical practice.

There are several studies explaining why NSE serves as an outcome predictor in cardiac arrest patients. Serum NSE increases in response to hypoxic brain injury, which can develop into hypoxic-ischemic encephalopathy [9,13,31,32]. It is possible that hypoxic-ischemic encephalopathy is more often the cause of death in younger patients compared to older patients during the immediate post-cardiac arrest phase, which could explain a potentially enhanced prognostic performance in younger individuals [35,36,37]. Furthermore, we cannot rule out that this association simply stems from a time-dilution effect, since overall mortality is generally higher for older cardiac arrest survivors [2,6]. In summary, we believe that NSE levels measured three days after cardiac arrest might be helpful to inform relatives regarding short-term and long-term prognosis. This might also facilitate decision making on whether to proceed with life-supporting measures or to establish a palliative approach.

There are limitations to this report. We did not measure NSE values on day four post-cardiac arrest and only measured NSE in patients during their ICU stay. We did not account for hemolysis, use of ventricular assist devices, extracorporeal life support, transfusions, kidney function or dialysis, which can all impact NSE values. Our sample size in the subgroups was relatively small, and age-related associations may have been diluted by comorbidities in the elderly patients. Additionally, we performed the neurological follow-up by telephone, which is likely inferior to in-person assessment. In addition, the CPC scale does not capture nuances of the neuropsychological sequalae often associated with cardiac arrest [38,39]. Additionally, we had no data concerning the cause of death. Finally, there is the issue of selection bias and self-fulfilling prophecy in neurological-prognostication research when having unblinded physicians. However, as NSE is used in clinical routines in our facility, blinding physicians to NSE values would be impractical due to ethical considerations.

## 5. Conclusions

In conclusion, NSE serum concentration measured three days after cardiac arrest is associated with long-term mortality and neurological outcome and may thus provide prognostic information that improves clinical decision making, particularly in the subgroup of younger patients <54 years, where NSE showed an excellent negative predictive value.

## Figures and Tables

**Table 1:** Baseline characteristics.

Factor	All Patients	Survivor	Non-Survivor	*p*-Value
Number (% of total)	403 (100)	176 (43.7)	227 (56.3)	
**Sociodemographics**				
Age, years, mean (SD)	64.1 (14.6)	59.7 (14.4)	67.6 (13.8)	<0.001
Male gender, n (%)	284 (70.5)	138 (78.4)	146 (64.3)	0.002
**Comorbidities (n, %)**				
Coronary artery disease	260 (64.8)	129 (73.7)	131 (58.0)	0.001
Congestive heart failure	56 (14.0)	16 (9.1)	40 (17.7)	0.014
COPD	34 (8.5)	4 (2.3)	30 (13.3)	<0.001
Hypertension	203 (50.6)	85 (48.6)	118 (52.2)	0.47
Diabetes mellitus	86 (21.4)	28 (16.0)	58 (25.7)	0.019
Chronic kidney disease	54 (13.5)	13 (7.4)	41 (18.1)	0.002
Malignant disease	44 (11.0)	9 (5.1)	35 (15.5)	0.001
Neurological disease	47 (11.7)	14 (8.0)	33 (14.6)	0.042
**Resuscitation measures**				
Time until ROSC, mean (SD)	22.3 (16.7)	17.3 (15.3)	26.4 (16.7)	<0.001
Observed cardiac arrest, n (%)	330 (82.3)	162 (92.0)	168 (74.7)	<0.001
Bystander CPR, n (%)	264 (65.7)	140 (79.5)	124 (54.9)	<0.001
**Setting of cardiac arrest (n, %)**				
At home	151 (38.7)	44 (26.2)	107 (48.2)	<0.001
Public	177 (45.4)	97 (57.7)	80 (36.0)	
IHCA	62 (15.9)	27 (16.1)	35 (15.8)	
**Initial rhythm (n, %)**				
Ventricular tachycardia	19 (4.7)	9 (5.1)	10 (4.4)	<0.001
Ventricular fibrillation	206 (51.2)	123 (69.9)	83 (36.7)	
Asystole	62 (15.4)	8 (4.5)	54 (23.9)	
Pulseless electrical activity	84 (20.9)	16 (9.1)	68 (30.1)	
Unknown	31 (7.7)	20 (11.4)	11 (4.9)	
**Diagnostic measures (mean, SD)**				
Initial lactate (mmol/L)	6.6 (4.3)	5.1 (3.3)	7.7 (4.6)	<0.001
Initial pH	7.2 (0.2)	7.3 (0.1)	7.2 (0.2)	0.003
**Cause of cardiac arrest (n, %)**				
Coronary artery disease	199 (49.4)	117 (66.5)	82 (36.1)	<0.001
Arrhythmia	70 (17.4)	26 (14.8)	44 (19.4)	0.23
Respiratory	67 (16.6)	13 (7.4)	54 (23.8)	<0.001
Other/unknown	67 (16.6)	20 (11.4)	47 (20.7)	0.012

Data presented as n (%) for binary and categorical variables and mean (SD) for continuous variables. Percentages indicate the proportion of a variable in relation to all patients, survivors and non-survivors. COPD, chronic obstructive pulmonary disease; ROSC, return of spontaneous circulation; CPR, cardiopulmonary resuscitation; IHCA, in-hospital cardiac arrest.

**Table 2:** (**a**) Association between NSE serum levels and long-term mortality. (**b**) Association between NSE serum levels and long-term neurological outcome.

(a) Primary Endpoint: Long-Term Mortality									
	N	All	Survivors	Non-Survivors	*p*-Value	Univariable HR (95%CI), *p*-Value	Multivariable * Adjusted HR (95%CI), *p*-Value	Multivariable ** Adjusted HR (95%CI), *p*-Value	ROC AUC (95% CI)
			n = 176	n = 227					
NSE max, median (IQR)	403	43.8 (27.6, 108.6)	29.7 (22.9, 45.7)	75.7 (35.2, 197.7)	<0.001	1.29 (1.23, 1.36), *p* < 0.001	1.32 (1.25, 1.39), *p* < 0.001	1.24 (1.17, 1.31), *p* < 0.001	0.77 (0.73, 0.82)
NSE day 0, median (IQR)	325	33.8 (24.3, 49.4)	28.6 (21.5, 41.8)	36.8 (27.6, 59.9)	<0.001	1.14 (1.08, 1.2), *p* < 0.001	1.16 (1.1, 1.23), *p* < 0.001	1.07 (1, 1.13), *p* = 0.038	0.64 (0.58, 0.70)
NSE day 1, median (IQR)	346	33.4 (23.4, 63.9)	26.9 (19.3, 37.3)	50.1 (29.8, 105)	<0.001	1.27 (1.2, 1.34), *p* < 0.001	1.3 (1.22, 1.38), *p* < 0.001	1.24 (1.15, 1.32), *p* < 0.001	0.74 (0.69, 0.80)
NSE day 2, median (IQR)	294	29.9 (19.5, 94.1)	22.3 (17.4, 30.6)	76.5 (29.8, 192)	<0.001	1.43 (1.33, 1.54), *p* < 0.001	1.47 (1.36, 1.6), *p* < 0.001	1.42 (1.31, 1.55), *p* < 0.001	0.8 (0.74, 0.85)
NSE day 3, median (IQR)	225	27.8 (17.4, 95.6)	19.8 (15.7, 27.8)	72.6 (26, 194)	<0.001	1.5 (1.38, 1.64), *p* < 0.001	1.55 (1.41, 1.71), *p* < 0.001	1.51 (1.37, 1.66), *p* < 0.001	0.81 (0.75, 0.87)
**(b) Secondary Endpoint: Neurological Outcome (CPC) after 2 Years**									
	**N**	**All**	**Good Neurological Outcome** **(CPC 1–2)**	**Poor Neurological Outcome** **(CPC 3–5)**	***p*-Value**	**Univariable** **OR (95%CI), *p*-Value**	Multivariable * Adjusted **OR (95%CI), *p*-Value**	Multivariable ** Adjusted **OR (95%CI), *p*-Value**	**ROC AUC** **(95%CI)**
			**n = 88**	**n = 215**					
NSE max, median (IQR)	303	55.9 (29.8, 148.9)	29.8 (21.35, 46.5)	80.1 (36.3, 204.8)	<0.001	1.45 (1.31, 1.61), *p* < 0.001	1.68 (1.45, 1.94), *p* < 0.001	1.41 (1.22, 1.62), *p* < 0.001	0.78 (0.73, 0.84)
NSE day 0, median (IQR)	247	35.4 (24.6, 54.7)	30.1 (21.1, 41.6)	37.7 (28, 65.1)	<0.001	1.19 (1.08, 1.32), *p* = 0.001	1.33 (1.16, 1.51), *p* < 0.001	1.09 (0.95, 1.24), *p* = 0.223	0.65 (0.57, 0.73)
NSE day 1, median (IQR)	256	38.4 (25.8, 76.1)	26.9 (16.7, 36.7)	53.9 (29.9, 109)	<0.001	1.41 (1.27, 1.57), *p* < 0.001	1.58 (1.37, 1.82), *p* < 0.001	1.35 (1.17, 1.55), *p* < 0.001	0.76 (0.70, 0.82)
NSE day 2, median (IQR)	208	35.5 (22.1, 137)	22.7 (17.3, 30.5)	87.8 (30.4, 212)	<0.001	1.52 (1.34, 1.73), *p* < 0.001	1.69 (1.42, 2.01), *p* < 0.001	1.53 (1.29, 1.81), *p* < 0.001	0.81 (0.75, 0.87)
NSE day 3, median (IQR)	167	38.5 (19.7, 134)	19.5 (14.5, 26.2)	87.1 (33.2, 200)	<0.001	1.68 (1.43, 1.96), *p* < 0.001	1.81 (1.49, 2.2), *p* < 0.001	1.67 (1.37, 2.03), *p* < 0.001	0.85 (0.79, 0.91)

(**a**) Comparison between NSE serum levels on different days to predict overall long-term mortality. Data presented as median (interquartile range) or mean (95% confidence interval). NSE, neuron specific enolase; HR, hazard ratio; ROC, receiver operating characteristics curve; AUC, area under the curve; IQR, interquartile range. * Adjusted for gender, age and comorbidities (coronary artery disease, congestive heart failure, COPD, hypertension, diabetes mellitus, chronic kidney disease, malignant disease, neurological disease). ** Adjusted for gender, age and resuscitation circumstances (ROSC, setting of cardiac arrest, bystander CPR, initial rhythm, initial lactate). (**b**) Comparison between NSE serum levels on different days to predict neurological outcome after two years. Data presented as median (interquartile range) or mean (95% confidence interval). NSE, neuron specific enolase; CPC, cerebral performance category; OR, odds ratio; ROC, receiver operating characteristics curve; AUC, area under the curve; IQR, interquartile range. * Adjusted for gender, age and comorbidities (coronary artery disease, congestive heart failure, COPD, hypertension, diabetes mellitus, chronic kidney disease, malignant disease, neurological disease). ** Adjusted for gender, age and resuscitation circumstances (ROSC, setting of cardiac arrest, bystander CPR, initial rhythm, initial lactate).

**Table 3:** Performance of NSE serum levels at different cut-off points to predict overall long-term mortality.

Max. NSE	Survivors below Cut-off (n)	Survivors above Cut-off (n)	Non-Survivors below Cut-off (n)	Non-Survivors above Cut-off (n)	Sensitivity Pr(+A), % (95%CI)	Specificity Pr(-N), % (95%CI)	ROC Area, (95%CI)	Likelihood Ratio (+), (95%CI)	Likelihood Ratio (−), (95%CI)	Hazard Ratio (95%CI)	PPV, % (95%CI)	NPV, % (95%CI)
33 ng/mL	100	76	48	179	78.9 (51.3–61.2)	56.8 (73.0–84.0)	0.68 (0.63–0.72)	1.83 (1.52–2.19)	0.37 (0.28–0.49)	4.91 (3.17–7.58)	70.2 (64.2–75.7)	67.6 (59.4–75.0)
45.9 ng/mL	133	43	77	150	66.1 (59.5–72.2)	75.6 (68.5–81.7)	0.71 (0.66–0.75)	2.70 (2.05–3.56)	0.45 (0.37–0.55)	6.03 (3.88–9.35)	77.7 (71.2–83.4)	63.3 (56.4–69.9)
Day 0 NSE												
33 ng/mL	85	51	76	113	59.8 (52.4–66.8)	62.5 (53.8–70.6)	0.61 (0.56–0.67)	1.59 (1.25–2.04)	0.64 (0.52–0.80)	2.48 (1.58–3.89)	68.9 (61.2–75.9)	52.8 (44.8–60.7)
32.9 ng/mL	85	51	74	115	60.8 (53.5–67.8)	62.5 (53.8–70.6)	0.62 (0.56–0.67)	1.62 (1.27–2.07)	0.63 (0.50–0.67)	2.59 (1.65–4.07)	69.3 (61.7–76.2)	53.5 (45.4–61.4)
Day 1 NSE												
33 ng/mL	110	49	62	125	66.8 (59.6–73.5)	69.2 (61.4–76.3)	0.68 (0.63–0.73)	2.17 (1.68–2.80)	0.48 (0.38–0.60)	4.53 (2.88–7.12)	71.8 (64.5–78.4)	64.0 (56.3–71.1)
31.1 ng/mL	105	54	53	134	71.7 (64.6–78.0)	66.0 (58.1–73.4)	0.69 (0.64–0.74)	2.11 (1.67–2.67)	0.43 (0.33–0.55)	4.92 (3.12–7.76)	71.3 (64.2–77.6)	66.5 (58.5–73.8)
Day 2 NSE												
33 ng/mL	121	28	46	99	68.3 (60.0–75.7)	81.2 (74.0–87.1)	0.75 (0.70–0.80)	3.63 (2.56–5.16)	0.39 (0.30–0.50)	9.30 (5.43–15.92)	78.0 (69.7–84.8)	72.5 (65.0–79.1)
29.5 ng/mL	110	39	36	109	75.2 (67.3–82.0)	73.8 (66.0–80.7)	0.74 (0.70–0.79)	2.87 (2.16–3.82)	0.34 (0.25–0.45)	8.54 (5.06–14.41)	73.6 (65.8–80.5)	75.3 (67.5–82.1)
Day 3 NSE												
33 ng/mL	89	22	34	80	70.2 (60.9–78.4)	80.2 (71.5–87.1)	0.75 (0.70–0.81)	3.54 (2.39–5.24)	0.37 (0.28–0.50)	9.52 (5.16–17.56)	78.4 (69.2–86.0)	72.4 (63.6–80.0)
35.9 ng/mL	94	17	36	78	68.4 (59.1–76.8)	84.7 (76.6–90.8)	0.77 (0.71–0.82)	4.47 (2.83–7.04)	0.37 (0.28–0.49)	11.98 (6.28–22.85)	82.1 (72.9–89.2)	72.3 (63.8–79.8)

At each day cut-off at 33 ng/mL as recommended and cut-off based on Youden index is shown. NSE, Neuron specific enolase; ROC, receiver operating curve; PPV, positive predictive value; NPV, negative predictive value.

## Supplementary Materials

The following are available online at https://www.mdpi.com/article/10.3390/medicines8110072/s1, First, we attached the comparison between NSE on day 5 and 7 and outcomes (Supplementary Table S1: Association between NSE serum levels at day 5 and 7 and long-term outcomes). Second, we attached an analysis for performance of NSE measurements at day 3 with a cut-off of 33 ng/mL for different points of follow-up-time (i.e., 6, 12, 18 and 24 months) concerning all-cause mortality (Supplementary Table S2: Performance of NSE serum levels at day 3 to predict mortality at different time points). We also attached an analysis for performance of NSE measurements at day 3 using a cut-off of 60 ng/mL for overall all-cause mortality and neurological outcome at 2 years (Supplementary Table S3: Performance of NSE serum levels at cut-off 60 ng/mL at day 3 to predict long-term outcomes). Additionally, we attached performance of NSE measurements on day 5 and 7 concerning long-term mortality (Supplementary Table S4: Performance of NSE serum levels on day 5 and day 7 at different cut-off points to predict overall long-term mortality) and concerning long-term neurological outcome on NSE from all available time points (Supplementary Table S5e: Performance of NSE serum levels at different cut-off points to predict neurological outcome after two years). Lastly, we attached an analysis of the performance of NSE in younger patients to predict overall long-term mortality at a cut-off of 33 ng/mL (Supplementary Table S6: Performance of NSE serum levels to predict overall long-term mortality in patients aged 16–53.9 years).
