# Supplementary material for: Neuron-Specific Enolase (NSE) Predicts Long-Term Mortality in Adult Patients after Cardiac Arrest: Results from a Prospective Trial
Source: Medicines (Basel). 2021 Nov 17;8(11):72. doi: 10.3390/medicines8110072 (PMC8624292; doi:10.3390/medicines8110072)
Supplement: Supplementary file 1 [file medicines-08-00072-s001.zip › medicines-1363421 supp.pdf]

# Supplementary Materials: Neuron-Specific Enolase (NSE) Predicts Long-Term Mortality in Adult Patients after Cardiac Arrest: Results from a Prospective Trial

**Jonas Müller, Benjamin Bissmann, Christoph Becker, Katharina Beck, Nina Loretz, Sebastian Gross, Simon A. Amacher, Chantal Bohren, Hans Pargger, Kai Tisljar, Raoul Sutter, Stephan Marsch and Sabina Hunziker**

**Table S1**: Association between NSE serum levels at day 5 & 7 and long-term outcomes.

| **Primary endpoint: Long-term mortality** | | | | | | | | | |
|---|---|---|---|---|---|---|---|---|---|
| | **N** | **All** | **Survivors** | **Non-Survivors** | **p-value** | **Univariable HR (95%CI), p-value** | **Multivariable* adjusted HR (95%CI), p-value** | **Multivariable** adjusted HR (95%CI), p-value** | **ROC AUC (95% CI)** |
| **NSE day 5, median (IQR)** | 122 | 22.9 (15.4, 47.5) | 17.7 (13.8, 25.5) | 40.1 (18.8, 84.3) | <0.001 | 1.42 (1.27, 1.59), p<0.001 | 1.42 (1.26, 1.6), p<0.001 | 1.44 (1.26, 1.65), p<0.001 | 0.76 (0.68, 0.85) |
| **NSE day 7, median (IQR)** | 78 | 21.4 (14.4, 33.3) | 20 (14.1, 26.3) | 24.9 (15.4, 45) | 0.040 | 1.14 (1.01, 1.29), p=0.028 | 1.17 (0.98, 1.39), p=0.076 | 1.18 (1.05, 1.33), p=0.007 | 0.64 (0.51, 0.76) |
| **Secondary endpoint: Neurological outcome (CPC) after 2 years** | | | | | | | | | |
| | **N** | **All** | **Good neurological outcome (CPC 1-2)** | **Poor neurological outcome (CPC 3-5)** | **p-value** | **Univariable (OR (95%CI), p-value)** | **Multivariable* adjusted OR (95%CI), p-value** | **Multivariable** adjusted OR (95%CI), p-value** | **ROC AUC (95%CI)** |
| **NSE day 5, median (IQR)** | 93 | 25.3 (17.3, 65.9) | 17.8 (14.1, 23.1) | 46 (22.4, 91.7) | <0.001 | 1.55 (1.27, 1.88), p<0.001 | 1.7 (1.3, 2.22), p<0.001 | 1.52 (1.19, 1.93), p=0.001 | 0.81 (0.72, 0.89) |
| **NSE day 7, median (IQR)** | 57 | 22.6 (15.6, 34.5) | 19.3 (14.3, 27.4) | 26.2 (17.4, 42.7) | 0.068 | 1.19 (0.97, 1.46), p=0.095 | 1.37 (0.96, 1.96), P=0.086 | 1.21 (0.95, 1.54), p=0.121 | 0.65 (0.50, 0.80) |

Data presented as median (interquartile range) or mean (95% confidence interval). NSE, neuron specific enolase; CPC, cerebral performance category; HR, hazard ratio; OR, odds ratio; ROC, receiver operating characteristics curve; AUC, area under the curve; IQR interquartile range. * adjusted for gender, age and comorbidities (coronary artery disease, congestive heart failure, COPD, hypertension, diabetes mellitus, chronic kidney disease, malignant disease, neurological disease). ** adjusted for gender, age and resuscitation circumstances (ROSC, setting of cardiac arrest, bystander CPR, initial rhythm, initial lactate).

**Table S2**: Performance of NSE serum levels at day 3 to predict mortality at different time points.

| Mortality | Survivors below cutoff (n) | Survivors above cutoff (n) | Non-survivors below cutoff (n) | Non-survivors above cutoff (n) | Sensitivity Pr(+A), % (95%CI) | Specificity Pr(-N), % (95%CI) | ROC area, (95%CI) | Likelihood ratio (+), (95%CI) | Likelihood ratio (-), (95%CI) | PPV, % (95%CI) | NPV, % (95%CI) |
|---|---|---|---|---|---|---|---|---|---|---|---|
| **at 6 month** | 100 | 26 | 23 | 76 | 76.8% (67.2%-84.7%) | 79.4% (71.2%-86.1%) | 0.78 (0.73 - 0.84) | 3.72 (2.60-5.33) | 0.29 (0.20-0.42) | 74.5% (64.9-82.6%) | 81.3% (73.3-87.8%) |
| **at 12 month** | 99 | 26 | 24 | 76 | 76.0% (66.4%-84.0%) | 79.2% (71.0%-85.9%) | 0.78 (0.72-0.83) | 3.65 (2.55-5.23) | 0.30 (0.21-0.43) | 74.5% (64.9%-82.6%) | 80.5% (72.4%-87.1%) |
| **at 18 month** | 99 | 26 | 24 | 76 | 76.0% (66.4%-84.0%) | 79.2% (71.0%-85.9%) | 0.78 (0.72-0.83) | 3.65 (2.55-5.23) | 0.30 (0.21-0.43) | 74.5% (64.9%-82.6%) | 80.5% (72.4%-87.1%) |
| **at 24 month** | 98 | 26 | 25 | 76 | 75.2% (65.7%-83.3%) | 79.0% (70.8%-85.8%) | 0.77 (0.72-0.83) | 3.59 (2.50-5.14) | 0.31 (0.22-0.45) | 74.5% (64.9%-82.6%) | 79.7% (71.5%-86.4%) |

**Table S3**: Performance of NSE serum levels at cut-off 60ng/mL at day 3 to predict long-term outcomes.

| | Overall long-term mortality | | | | | | | | | | | |
|---|---|---|---|---|---|---|---|---|---|---|---|---|
| | **Survivors below cutoff (n)** | **Survivors above cutoff (n)** | **Non-survivors below cutoff (n)** | **Non-survivors above cutoff (n)** | **Sensitivity Pr(+A), % (95%CI)** | **Specificity Pr(-N), % (95%CI)** | **ROC area, (95%CI)** | **Likelihood ratio (+), (95%CI)** | **Likelihood ratio (-), (95%CI)** | **Hazard ratio (95%CI)** | **PPV, % (95%CI)** | **NPV, % (95%CI)** |
| NSE 60 ng/mL | 105 | 6 | 53 | 61 | 53.5% (43.9%-62.9%) | 94.6% (88.6%-98.0%) | 0.74 (0.69-0.79) | 9.90 (4.46-21.96) | 0.49 (0.40-0.60) | 20.14 (8.35-48.39) | 91.0% (81.5%-96.6%) | 66.5% (58.5%-73.8%) |
| | **CPC after 2 years** | | | | | | | | | | | |
| | **Good neurological outcome (CPC 1-2) below cutoff (n)** | **Good neurological outcome (CPC 1-2) above cutoff (n)** | **Poor neurological outcome (CPC 3-5) below cutoff (n)** | **Poor neurological outcome (CPC 3-5) above cutoff (n)** | **Sensitivity Pr(+A), % (95%CI)** | **Specificity Pr(-N), % (95%CI)** | **ROC area, (95%CI)** | **Likelihood ratio (+), (95%CI)** | **Likelihood ratio (-), (95%CI)** | **Odds ratio (95%CI)** | **PPV, % (95%CI)** | **NPV, % (95%CI)** |
| NSE 60 ng/mL | 59 | 3 | 44 | 61 | 58.1% (48.1%-67.7%) | 95.2% (86.5%-99.0%) | 0.77 (0.71-0.82) | 12.01 (3.93-36.64) | 0.44 (0.35-0.56) | 27.27 (8.47-86.94) | 95.3% (86.9%-99.0%) | 57.3% (47.2%-67.0%) |

Neuron specific enolase at a cut-off 60ng/mL day 3; ROC, receiver operating curve; PPV, positive predictive value; NPV, negative predictive value.

**Table S4**: Performance of NSE serum levels on day 5 & day 7 at different cutoff points to predict overall long-term mortality.

| | Survivors below cutoff (n) | Survivors above cutoff (n) | Non-survivors below cutoff (n) | Non-survivors above cutoff (n) | Sensitivity Pr(+A), % (95%CI) | Specificity Pr(-N), % (95%CI) | ROC area, (95%CI) | Likelihood ratio (+), (95%CI) | Likelihood ratio (-), (95%CI) | Hazard ratio (95%CI) | PPV, % (95%CI) | NPV, % (95%CI) |
|---|---|---|---|---|---|---|---|---|---|---|---|---|
| **Day 5 NSE** | | | | | | | | | | | | |
| 33 ng/mL | 54 | 9 | 26 | 33 | 55.9 (42.4-68.8) | 85.7 (74.6-93.3) | 0.71 (0.63-0.79) | 3.29 (2.05-7.47) | 0.51 (0.38-0.70) | 7.62 (3.21-17.98) | 78.6 (63.2-89.7) | 67.5 (56.1-77.6) |
| 27.4 ng/mL | 51 | 12 | 23 | 36 | 61.0 (47.4-73.5) | 81.0 (69.1-89.8) | 0.71 (0.63-0.79) | 3.20 (1.85-5.54) | 0.48 (0.34-0.68) | 6.65 (2.96-14.95) | 75.0 (60.4-86.4) | 68.9 (57.1-79.2) |
| **Day 7 NSE** | | | | | | | | | | | | |
| 33 ng/mL | 33 | 5 | 25 | 15 | 37.5 (22.7-54.2) | 86.8 (71.9-95.6) | 0.62 (0.53-0.72) | 2.85 (1.15-7.08) | 0.72 (0.55-0.94) | 3.96 (1.31-11.89) | 75.0 (50.9-91.3) | 56.9 (43.2-69.8) |
| 26.2 ng/mL | 28 | 10 | 21 | 19 | 47.5 (31.5-63.9) | 73.7 (56.9-86.6) | 0.61 (0.50-0.71) | 1.80 (0.97-3.37) | 0.71 (0.50-1.01) | 2.53 (0.99-6.48) | 65.5 (45.7-82.1) | 57.1 (42.2-71.2) |

At each day cutoff at 33 ng/mL as recommended and cutoff based on Youden index is shown. NSE Neuron specific enolase; ROC, receiver operating curve; PPV, positive predictive value; NPV, negative predictive value

**Table S5**:Performance of NSE serum levels at different cutoff points to predict neurological outcome after two years.

| | Good neurological outcome below cutoff (n) | Good neurological outcome above cutoff (n) | Poor neurological outcome below cutoff (n) | Poor neurological outcome above cutoff (n) | Sensitivity Pr(+A), % (95%CI) | Specificity Pr(-N), % (95%CI) | ROC area, (95%CI) | Likelihood ratio (+), (95%CI) | Likelihood ratio (-), (95%CI) | Odds ratio (95%CI) | PPV, % (95%CI) | NPV, % (95%CI) |
|---|---|---|---|---|---|---|---|---|---|---|---|---|
| **Max. NSE** | | | | | | | | | | | | |
| **33 ng/mL** | 49 | 39 | 45 | 170 | 79.1% (73.0-84.3%) | 55.7% (44.4-66.3%) | 0.67 (0.61-0.73) | 1.78 (1.4-2.28) | 0.38 (0.27-0.52) | 4.75 (2.79-8.08) | 81.3% (75.4-86.4%) | 52.1% (41.6-62.5%) |
| **42.1 ng/mL** | 64 | 24 | 63 | 152 | 70.7% (64.1-767%) | 72.7% (62.2-81.7%) | 0.72 (0.66-0.77) | 2.59 (1.82-3.69 | 0.40 (0.32-0.51) | 6.43 (3.71-11.16) | 86.4% (80.4-91.1%) | 50.4% (41.4-59.4%) |
| **Day 0 NSE** | | | | | | | | | | | | |
| **33 ng/mL** | 40 | 28 | 72 | 107 | 59.8% (52.2-67.0%) | 58.8% (46.2-70.6%) | 0.59 (0.52-0.66) | 1.45 (1.07-1.98) | 0.68 (0.52-0.89) | 2.12 (1.21-3.73) | 79.3% (71.4-85.8%) | 35.7% (26.9-45.3%) |
| **34.7 ng/mL** | 44 | 24 | 76 | 103 | 57.5% (49.9-64.9%) | 64.7% (52.2-75.9%) | 0.61 (0.54-0.68) | 1.63 (1.15-2.30) | 0.66 (0.51-0.84) | 2.48 (1.40-4.42) | 81.1% (73.2-87.5%) | 36.7% (28.1-45.9%) |
| **Day 1 NSE** | | | | | | | | | | | | |
| **33 ng/mL** | 54 | 25 | 56 | 121 | 68.4% (61.0-75.1%) | 68.4% (56.9-78.4%) | 0.68 (0.62-0.75) | 2.16 (1.54-3.03) | 0.46 (0.36-0.60) | 4.67 (2.65-8.23) | 82.9% (75.8-88.6%) | 49.1% (39.4-58.8%) |
| **38 ng/mL** | 62 | 17 | 66 | 111 | 62.7% (55.1-69.9%) | 78.5% (67.8-86.9%) | 0.71 (0.65-0.76) | 2.91 (1.88-4.51) | 0.48 (0.38-0.59) | 6.13 (3.32-11.31) | 86.7% (79.6-92.1%) | 48.4% (39.5-57.4%) |
| **Day 2 NSE** | | | | | | | | | | | | |
| **33 ng/mL** | 59 | 13 | 40 | 96 | 70.6% (62.2-78.1%) | 81.9% (71.1-90.0%) | 0.76 (0.70-0.82) | 3.91 (2.37-6.47) | 0.36 (0.27-0.48) | 10.89 (5.42-21.87) | 88.1% (80.5-93.5%) | 59.6% (49.3-69.3%) |
| **31.9 ng/mL** | 57 | 15 | 37 | 99 | 72.8% (64.5-80.1%) | 79.2% (68.0-87.8%) | 0.76 (0.70-0.82) | 3.49 (2.20-5.55) | 0.34 (0.25-0.46) | 10.17 (5.16-20.01) | 86.8% (79.2-92.4%) | 60.6% (50.0-70.6%) |
| **Day 3 NSE** | | | | | | | | | | | | |
| **33 ng/mL** | 52 | 10 | 26 | 79 | 75.2% (65.9-83.1%) | 83.9% (72.3-92.0%) | 0.80 (0.73-0.86) | 4.66 (2.62-8.32) | 0.30 (0.21-0.42) | 15.80 (7.09-35.1) | 88.8% (80.3-94.5%) | 66.7% (55.1-76.9%) |
| **37.1 ng/mL** | 54 | 8 | 28 | 77 | 73.3% (63.8-81.5%) | 87.1% (76.1-94.3%) | 0.80 (0.74-0.86) | 5.68 (2.95-10.96) | 0.31 (0.22-0.43) | 18.56 (7.95-43.15) | 90.6% (82.3-95.8%) | 65.9% (54.6-76.0%) |

| | | | | | | | | | | | |
|---|---|---|---|---|---|---|---|---|---|---|---|
| **Day 5 NSE** | | | | | | | | | | | |
| **33 ng/mL** | 34 | 3 | 22 | 34 | 60.7% (46.8-73.5%) | 91.9% (78.1-98.3%) | 0.76 (0.68-0.84) | 7.49 (2.48-22.61) | 0.43 (0.30-0.60) | 17.52 (5.04-59.81) | 91.9% (78.1-98.3%) | 60.7% (46.8-73.5%) |
| **25.7 ng/mL** | 32 | 5 | 18 | 38 | 67.9% (54.0-79.7%) | 86.5% (71.2-95.5%) | 0.77 (0.69-0.85) | 5.02 (2.18-11.57) | 0.37 (0.25-0.56) | 13.51 (4.62-39.15) | 88.4% (74.9-96.1%) | 64.0% (49.2-77.1%) |
| **Day 7 NSE** | | | | | | | | | | | |
| **33 ng/mL** | 17 | 3 | 23 | 14 | 37.8% (22.5-55.2%) | 85.0% (62.1-96.8%) | 0.61 (0.50-0.73) | 2.52 (0.82-7.75) | 0.73 (0.54-1.00) | 3.45 (0.9-12.88) | 82.4% (56.6-96.2%) | 42.5% (27.0-59.1%) |
| **21.7 ng/mL** | 13 | 7 | 15 | 22 | 59.5% (42.1-75.2%) | 65.0% (40.8-84.6%) | 0.62 (0.49-0.76) | 1.70 (0.88-3.27) | 0.62 (0.38-1.03) | 2.72 (0.90-8.24) | 75.9% (56.5-89.7%) | 44.1% (27.2-62.1%) |

At each day cutoff at 33 ng/mL as recommended and cutoff based on Youden index is shown. CPC 1-2 was considered good neurological outcome and CPC 3-5 as poor neurological outcome. NSE, neuron specific enolase; ROC, receiver operating curve; PPV, positive predictive value; NPV, negative predictive value.

**Table S6**: Performance of NSE serum levels to predict overall long-term mortality in patients aged 16-53.9 years.

| | | | Overall Mortality | | |
|---|---|---|---|---|---|
| **Max. NSE** | **Sensitivity Pr(+A), % (95%CI)** | **Specificity Pr(-N), % (95%CI)** | **ROC area, (95%CI)** | **PPV, % (95%CI)** | **NPV, % (95%CI)** |
| 33 ng/mL | 100.0% (88.8-100.0%) | 44.6% (31.3-58.5%) | 0.72 (0.66-0.79) | 50.0% (37.0-63.0%) | 100.0% (86.3-100.0%) |
| **Day 0 NSE** | | | | | |
| 33 ng/mL | 71.4% (51.3-86.8%) | 57.8% (42.2-72.3%) | 0.65 (0.53-0.76) | 51.3% (34.8-67.6%) | 76.5% (58.8-89.3%) |
| **Day 1 NSE** | | | | | |
| 33 ng/mL | 88.9% (70.8-97.6%) | 60.0% (45.9-73.0%) | 0.74 (0.66-0.83) | 52.2% (36.9-67.1%) | 91.7% (77.5-98.2%) |
| **Day 2 NSE** | | | | | |
| 33 ng/mL | 92.0% (74.0-99.0%) | 80.0% (66.3-90.0%) | 0.86 (0.78-0.94) | 69.7% (51.3-84.4%) | 95.2% (83.8-99.4%) |
| **Day 3 NSE** | | | | | |
| 33 ng/mL | 100.0% (83.9-100.0%) | 80.0% (65.4-90.4%) | 0.90 (0.84-0.96) | 70.0% (50.6-85.3%) | 100.0% (90.3-100.0%) |
| **Day 5 NSE** | | | | | |
| 33 ng/mL | 90.0% (55.5-99.7%) | 83.3% (62.6-95.3%) | 0.87 (0.74-0.99) | 69.2% (38.6-90.9%) | 95.2% (76.2-99.9%) |
| **Day 7 NSE** | | | | | |
| 33 ng/mL | 100.0% (29.2-100.0%) | 83.3% (58.6-96.4%) | 0.92 (0.83-1.00) | 50.0% (11.8-88.2%) | 100.0% (78.2-100.0%) |

Cutoff at 33 ng/mL as recommended is shown; NSE, neuron specific enolase; ROC, receiver operating curve; PPV, positive predictive value; NPV, negative predictive value.
